# Estimating Radiation Shielding of Fired Clay Bricks Using ANN and GEP Approaches

**DOI:** 10.3390/ma15175908

**Published:** 2022-08-26

**Authors:** Muhammad Nasir Amin, Izaz Ahmad, Asim Abbas, Kaffayatullah Khan, Muhammad Ghulam Qadir, Mudassir Iqbal, Abdullah Mohammad Abu-Arab, Anas Abdulalim Alabdullah

**Affiliations:** 1 Department of Civil and Environmental Engineering, College of Engineering, King Faisal University, Al-Ahsa 31982, Saudi Arabia; 2 Department of Civil Engineering, University of Engineering and Technology, Peshawar 25120, Pakistan; 3 Department of Environmental Sciences, Abbottabad Campus, COMSATS University Islamabad, Abbottabad 22060, Pakistan

**Keywords:** fired clay bricks, radiation shielding, compressive strength, artificial neural network, gene expression programming, parametric and sensitivity analysis

## Abstract

This study aimed to determine how radiation attenuation would change when the thickness, density, and compressive strength of clay bricks, modified with partial replacement of clay by fly ash, iron slag, and wood ash. To conduct this investigation, four distinct types of bricks—normal, fly ash-, iron slag-, and wood ash-incorporated bricks were prepared by replacing clay content with their variable percentages. Additionally, models for predicting the radiation-shielding ability of bricks were created using gene expression programming (GEP) and artificial neural networks (ANN). The addition of iron slag improved the density and compressive strength of bricks, thus increasing shielding capability against gamma radiation. In contrast, fly ash and wood ash decreased the density and compressive strength of burnt clay bricks, leading to low radiation shielding capability. Concerning the performance of the Artificial Intelligence models, the root mean square error (RMSE) was determined as 0.1166 and 0.1876 nC for the training and validation data of ANN, respectively. The training set values for the GEP model manifested an RMSE equal to 0.2949 nC, whereas the validation data produced RMSE = 0.3507 nC. According to the statistical analysis, the generated models showed strong concordance between experimental and projected findings. The ANN model, in contrast, outperformed the GEP model in terms of accuracy, producing the lowest values of RMSE. Moreover, the variables contributing towards shielding characteristics of bricks were studied using parametric and sensitivity analyses, which showed that the thickness and density of bricks are the most influential parameters. In addition, the mathematical equation generated from the GEP model denotes its significance such that it can be used to estimate the radiation shielding of burnt clay bricks in the future with ease.

## 1. Introduction

The application of radioactive materials in agriculture research, medicine, and power generation plays a vital role in the economic and technological development of a country. Major applications of nuclear technology include diagnosis and treatment of a variety of diseases, generation of electricity, archaeology, pollution mitigation, etc. [1,2,3,4,5,6,7,8,9]. Nuclear technology uses different radioactive rays, such as gamma rays, X-rays, and neutrons, that have the potential to cause serious health and environmental problems [10,11,12,13]. Due to the increase in the application of nuclear technology in the modern world, protecting humans and the whole living environment from the adverse effects of radiation represents vital and affirming protection against their harmful actions [14]. The hazardous effect of nuclear technology increases the global concern about providing shielding against radiation or controlling the use of radioactive materials. Most nuclear activities are carried out in an enclosed built environment. Thus, material selection for construction of the enclosed built environment is one of the major challenges in the field of nuclear technology. The literature has revealed that the shielding capability of materials against radiation depends on the density and thickness of the material [15]. Higher shielding against radiation can be achieved with materials that have a relatively high density such as barite, goethite, magnetite, hematite, serpentine, lead, heavy-metal oxide, steel slag, steel shot, and colemanite [16,17,18,19,20,21,22,23]. Higher-density materials have a significant impact on the reduction of thickness of structural members while providing remarkable performance against radiation [24,25,26,27,28,29,30,31]. In general, concrete is most widely used for building important radiation-containing structures such as radiation therapy chambers in hospitals, nuclear power stations, and particle accelerators to provide radiation shielding due to its higher relative density [32,33]. Numerous studies conducted on dense concrete show significant improvement of concrete performance against radiation and many other inherent advantages as compared to other materials [14,15,32,33,34,35]. On the other hand, denser materials may have adverse effects on the other properties of concrete, such as affecting compressive and tensile strengths, along with reduction in the elastic modulus [15,35]. Bricks are also used worldwide in the construction of different facilities, not limited to partitions and loading-bearing walls [25]. Brick walls provide better insulation properties than other building materials, which are also cost-effective, easy to obtain and use, and made of eco-friendly natural materials. Due to their mechanical and thermal properties, bricks can be a good alternative for radiation protection applications. Besides other characteristics, bricks have enough density and strength, which contributes to their radiation shielding feature [36]. Numerous researchers have experimentally investigated the shielding performance of different types of bricks [37,38,39,40,41], as listed in Table 1. For instance, Mann et al. [37] studied the response of bricks against radiation with different fly ash compositions at different photon energies. The results suggested that fly ash brick can be used for medium-energy photon attenuation. Mann et al. [42] investigated the usage of burnt clay brick for surface storage facilities subjected to 0.001–15 MeV gamma ray photon energies. The result suggested that clay bricks are suitable materials that provide environmentally safe storage facilities against radioactive emission at given photon energies. Kiatwattanacharoen et al. [43] studied the behavior of bricks consisting of barium sulphate against X-ray radiation. It was found in the results that the values of the half-value layer for barium sulphate bricks were lower compared to other types of bricks and concrete. Sayyed et al. [38] studied the shielding ability of various types of bricks against gamma rays and found that steel-slag brick considerably lowers the energy of gamma ray photons and thus provides a better shield against gamma rays. Escalera-Velasco et al. [40] recommended that artisanal bricks with high density provided more effective shielding capability for low-energy photons in comparison with gypsum. Velasco et al. [44] concluded that bricks can be used safely for the construction of medical facilities containing less than 30 keV mammography units. Durak et al. [36] determined that rising the level of Cobalt metal added improves the gamma- and neutron-shielding capacity of the brick samples. Sidhu et al. [45] reported that, based on his research work, that fly ash–lime–Gypsum bricks possess satisfactory radiation shielding properties and can be used as environmentally safe storage facilities for low levels of nuclear waste. Echeweozo et al. [46] studied Granite–Kaolin Composite Bricks and determined that brick samples prepared with Granite–Kaolin Composite were thermally stable, good in gamma radiation shielding, and efficient in liquid radioactive waste immobilization. El-khatib et al. [47] reported that clay materials would be good applicants for use in civil engineering construction as well as in the fabrication of building materials used in medical and nuclear facilities.

It is revealed from experimental studies that the radiation shielding capability of any material depends on its density and thickness. The radiation shielding phenomenon is a highly non-linear complex problem that may be related to material properties other than density and thickness. The already established relation for radiation shielding has an experimental dependency. This study aims to identify the most influential properties of shielding materials and to develop a reliable correlation considering mechanical properties of the shielding material. ANN and GEP are used in the study to consider the high nonlinearity of the problem [15,20,36,38,48,49,50,51,52]. It is also inferred that many experimental studies are available on the radiation shielding capabilities of fired clay bricks. However, no machine learning model exists on this topic. Moreover, AI techniques are widely used for solving engineering problems owing to their efficiency in avoiding laborious experimental work. Kavya et al. [53] developed an artificial neural network (ANN) model for predicting the strengths of concrete containing glass and basalt fibers. The results have shown that ANN has great potential to predict the compressive, split tensile, and flexural strengths of glass fiber-reinforced concrete and basalt fiber-reinforced concrete. Almashaqbeh et al. [54] predicted the post-heating mechanical properties of cementitious composites reinforced with multi-scale additives using an Artificial Neural Network (ANN) approach. The results showed that ANN models have strong potential to predict the mechanical properties of cementitious composites. Amin et al. [23] developed artificial intelligence computational models for concrete radiation ability using ANN and GEP approaches. The statistical evaluation revealed that AI models show close agreement between the experimental and predicted results. However, the ANN model yielded better accuracy than the GEP model, showing higher R and lower MAE and RMSE values. Khan et al. developed an AI model for the estimation of flexural strength capacity of FRP reinforced beams using Random Forest and Artificial Neural Network approaches. Both models showed acceptable results; however, the ANN model showed superior accuracy and performance compared to the Random Forest model. Yadollahi et al. [55] used an artificial neural network to predict the optimal mixture for radiation-shielding concrete. ANN was found to be better at achieving reliable results. It was determined with the help of ANN that the optimum mixture of radiation-shielding concrete has a water–cement ratio of 0.45, cement quantity of 390 kg, a volume fraction of lead slag aggregate of 60%, and a silica fume–cement ratio of 0.15. ANNs are generally considered to be robust prediction models in comparison to other AI models; however, it has a black box nature. GEP extracts nonlinear correlation among the variables in the form of simple mathematical equations [23,56,57]. Therefore, this study compared a black box model (ANN) with the easily determined mathematical equation-generating model (GEP) to identify the most influential properties of the shielding material and to develop a reliable correlation for the calculation of radiation shielding of bricks in terms of their mechanical properties.

## 2. Methodology

**Figure 1:**
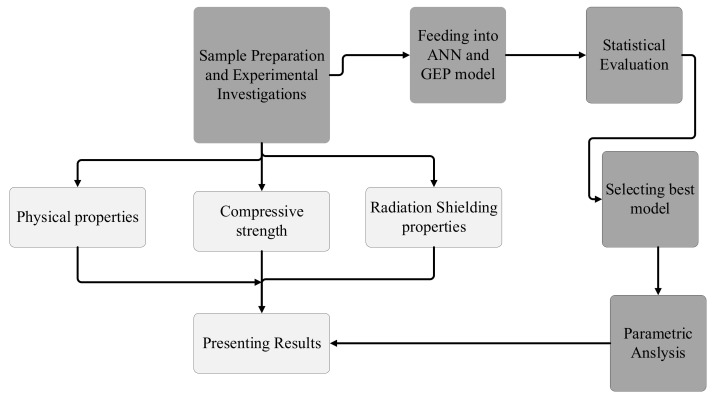
Flow diagram of the undertaken research.

This section describes the details of the materials used; specimen preparation; and experimental setup for physical, mechanical, and radiation-shielding properties. A brief introduction to artificial intelligence is also part of this section. As shown in Figure 1, samples were prepared for investigating their physical, mechanical, and radiation properties. Those properties were fed into the ANN and GEP models for developing prediction models. The statistical evaluation assessed the performance of the models to select the best model. The selected model was used to validate the findings using parametric analysis.

### 2.1. Specimen Description

**Figure 2:**
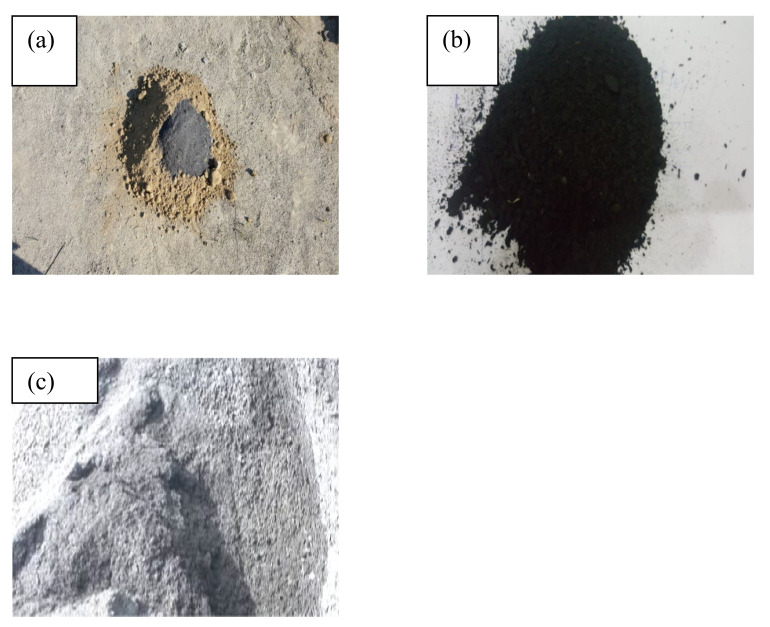
(**a**) Iron slag added to clay. (**b**) Fly ash sample. (**c**) Wood ash sample.

Four types of bricks—(1) conventional fired clay bricks, (2) clay–fly ash bricks, (3) clay–iron slag bricks, and (4) clay–wood ash bricks—were investigated in the study to evaluate for their radiation-shielding ability. The fly ash, wood ash, and iron slag samples are shown in Figure 2. The bricks studied were manufactured as per the standard practice of ASTM C-67. Wood ash used in the study was collected locally as a byproduct of domestic and commercial wood burning. Fly ash and iron slag were obtained from the local market. The percentage replacements of the added materials are listed in Table 2. All materials were added as a replacement of clay by weight. The physical properties (ASTM D7263-21/ASTM D 854-02), chemical composition (ASTM-D4326), and grain size distribution (ASTM D 422-63/ASTM D7928-21) of the constituent materials are listed in Table 3, Table 4 and Table 5, respectively.

### 2.2. Specimen Preparation

**Figure 3:**
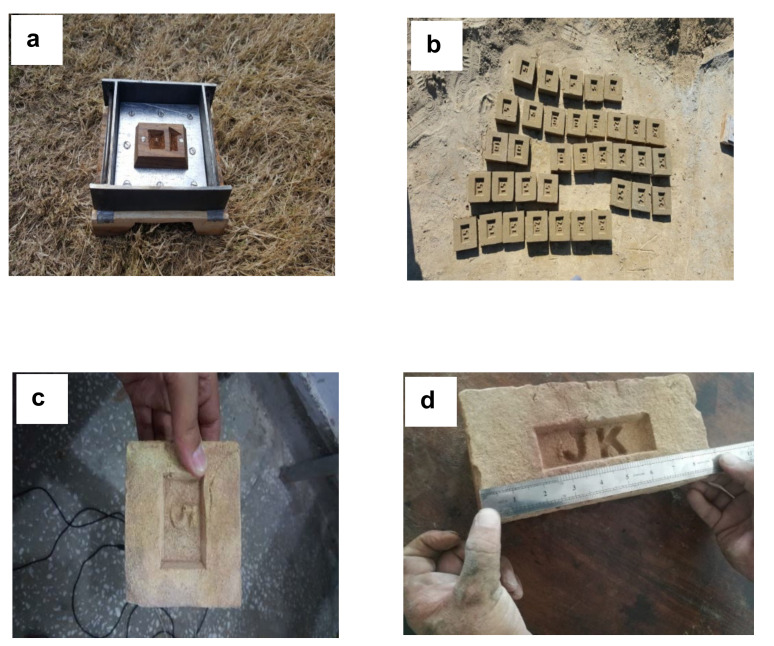
(**a**) Mold of radiation specimen. (**b**) Radiation brick specimen preparation. (**c**) Radiation brick specimen. (**d**) Normal brick specimen.

Brick specimens were prepared as per the standard practice of ASTM C 62. Brick specimens were prepared in standard dimensions (4.5 cm × 9 cm × 9 cm) for the determination of mechanical and physical properties. In comparison, square brick specimens (10 cm × 10 cm) were prepared for radiation shielding, with thicknesses of 2, 4, 6, 8, and 10 cm. Figure 3 presents the specimen and mold for the experimental setup.

### 2.3. Test Conducted on Brick Specimens

Brick specimens were subjected to different tests for the determination of physical, mechanical, and radiation-shielding properties.

#### 2.3.1. Density Determination

The density of the bricks was determined as per ASTM C20. This test method is the preferred choice for quality control and research purposes. Specimens are first oven dried and weighted (*D*). Then, the samples are immersed in water and boiled for 2 h. The specimens are cooled to room temperature while still immersed in water, and the suspended weights (S) of specimens are determined. The specimens are brought into a saturated surface dry state, and saturated weight (*W*) is determined. The bulk density (*B*) is calculated using the following equation:

$$B=\frac{D}{V}$$

where *V* = *W* − *D*

#### 2.3.2. Compressive Strength Test

**Figure 4:**
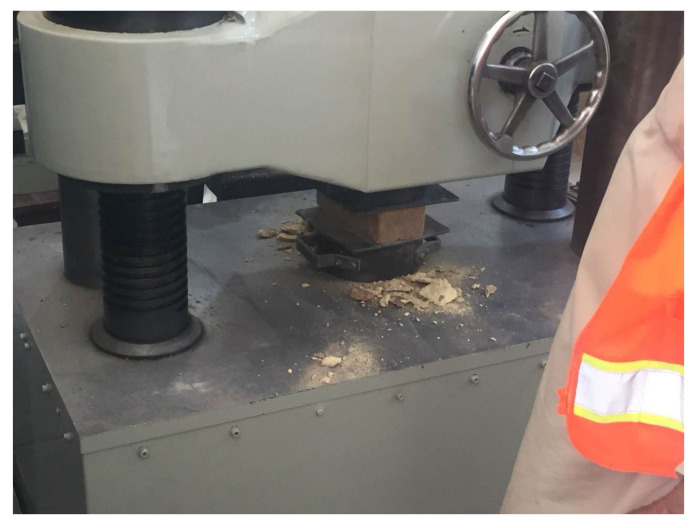
Compressive strength in universal testing machine.

Standard brick specimens were subjected to a compressive strength test as per ASTM C67. The test specimens consisted of half brick units that had been dried and cooled with full width and height. The test specimens are subjected to compressive force till failure in the universal test machine, i.e., the crushing of bricks as shown in Figure 4. The crushing load (P) is divided by the half brick area (A) to calculate the compressive strength of the bricks.

#### 2.3.3. Radiation Testing of Bricks

The Linear attenuation coefficient, which is a measure of radiation shielding ability, was measured to find the radiation ability of brick specimens. The test was performed in a Theratron Phoenix machine as per ASTM C1831/C1831M-17, in a cancer treatment hospital (IRNUM Hospital Peshawar, Pakistan) using Cobalt-60 as a Gamma-ray source. The test setup consisted of the following steps:

- Measuring of Gamma ray intensity (*N_o_*) by the detector when no brick specimens were placed between the source and detector.
- Measuring of Gamma ray intensity (*N*) by the detector when brick specimens were placed between the source and detector.

The linear attenuation coefficient was then measured using the following equation.

$$\;µ=\frac{1}{x}ln\frac{N_{○}}{N}\;\;$$

where

μ = Linear attenuation coefficient
*x* = material thickness in cm

### 2.4. Algorithms Adopted for the Development of AI Models

To evaluate the radiation shielding ability of fired clay bricks, this study was designed to use artificial neural networks (ANNs) and gene expression programming (GEP). This section provides an overview of the machine learning modelling strategies used in this study.

#### 2.4.1. Artificial Neutral Network Modelling

ANNs are inspired by the human brain and works similarly to biological neuron signals [58,59,60]. Neural networks depend on training data to learn and improve their performance and accuracy over time. Once these algorithms are fine-tuned, they can then be used for accurate prediction of a phenomenon. ANNs are considered simple mathematical models for enhancing existing data analysis technologies. ANNs are a mathematical and computational method employed to simulate the interdependencies between the input and output variable(s). The most commonly used type of ANN is the multilayer perceptron (MLP), which consists of an input layer, hidden layers (maybe more than one), and output layers. A neuron in a single layer has many parameters, but these parameters have no relation. The number of neurons required for the input and output layer depends on the variables in the input and output layer [61]. It is the hidden layer where computation takes place, and the number of neurons is required to be known for a suitable response to be achieved. To train the model, data is introduced into the input and output layer, and an appropriate model is created. The weights and biases of the model are adjusted to achieve the minimum error by calculating the difference between the output predicted values and actual values [62]. An optimized ANN model is obtained by changing the number of neurons in the hidden layer. The Lavenberg–Marquardt function was used to optimize the weights and performance of the network because it is considered to be the best function for the training of supervised algorithms [63]. Purelin and tan-sigmoid functions were used for activation both in the output and input layers, respectively. For statistical evaluation, Mean Absolute Error (MAE), Root Mean Square Error (RMSE), coefficient of correlation (R), and coefficient of determination (R^2^) were calculated following previous studies [64,65,66,67].

The experimental database employed for the ANN model is presented in Table 6 and Table 7. Four types of bricks—type 1 (conventional brick), type II (clay–fly ash bricks), type III (clay–iron slag bricks), and type IV (clay–wood ash bricks)—were used in the study. Appendix A contains ANN code used in modelling.

#### 2.4.2. GEP Modelling

GEP is a modern evolutionary algorithm for the development of AI models. GEP models can learn and adapt, much like living organisms, by changing their shape, size, and composition. Gene Expression Programming (GEP) is capable of automatically creating computer programs. These computer programs can be conventional mathematical models, decision trees, non-linear regression models, logistic regression models, and so on. Despite their complex nature, all GEP models are encoded in very simple linear structures. These chromosomes can mutate and reproduce the best one to create better programs. The chromosome that produces the greatest outcomes is passed down to the succeeding generations, and the process continues until an acceptable fitness level is achieved [68]. GEP has been used in several engineering applications to develop the prediction of different concrete properties for different types of concrete. GEP is a powerful and valuable technique for the development of prediction models. [23,57,69].

**Figure 5:**
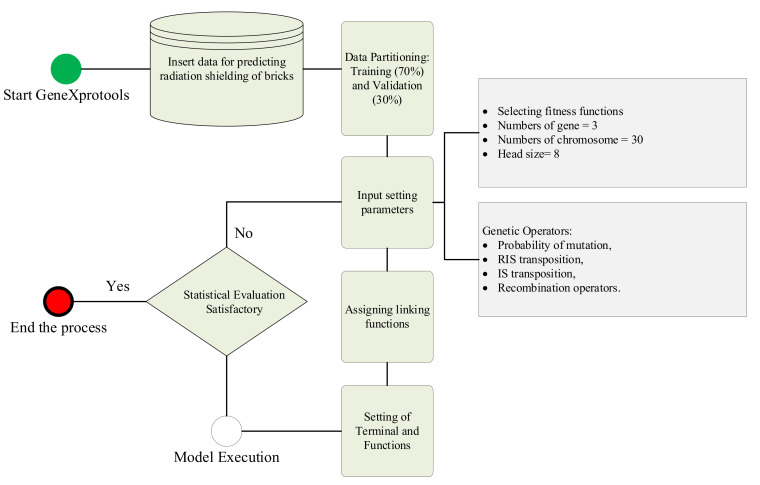
Schematics of GEP modelling.

The experimental data shown in Table 6 and Table 7 were used as training and validation in GeneXprotools. The purpose of using GEP was to develop a mathematical model that can be used later for computation purposes of radiation-shielding ability. The schematic diagram for GEP modelling is shown in Figure 5. Trials were carried out on setting parameters such as gene and chromosome numbers and head size, and finally, 3 genes, 30 chromosomes, and 10 head sizes were selected as hyperparameters, as they resulted in the best model.

## 3. Results and Discussion

### 3.1. Experimental Results

**Figure 6:**
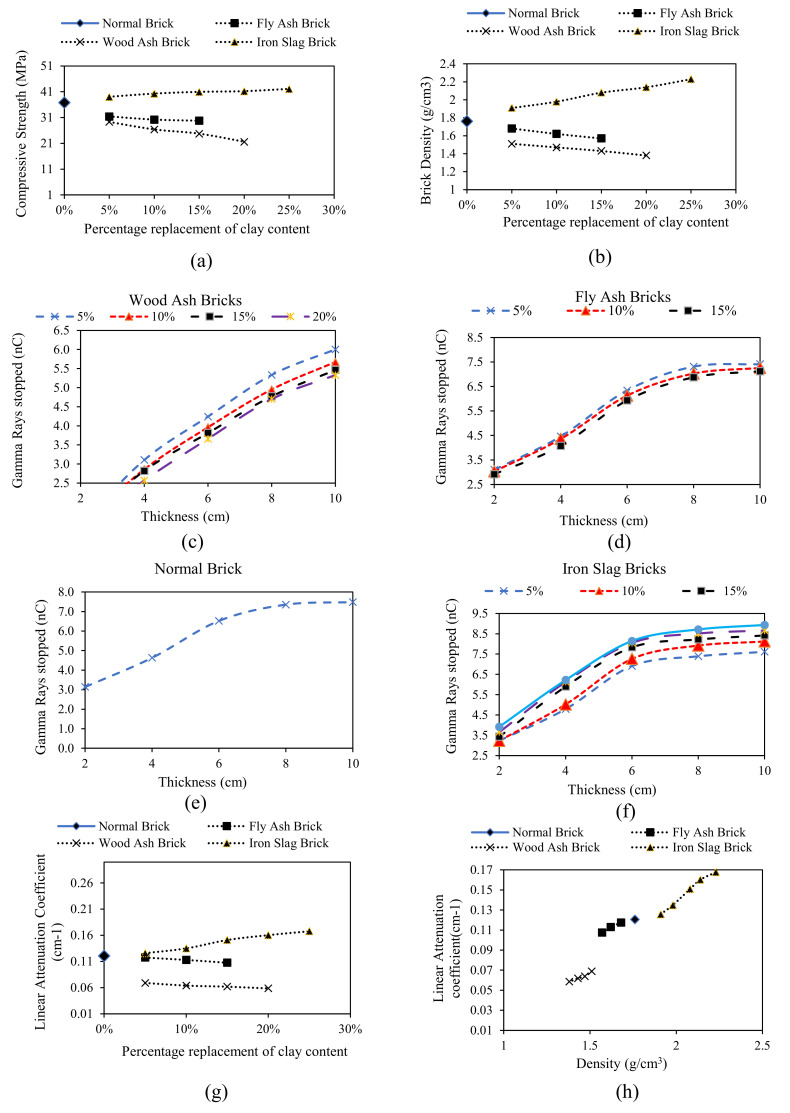
Experimental test results.

Results of compressive strength tests are presented in Figure 6a. The addition of iron slag as a replacement for clay has positive effects on compressive strength compared to the conventional brick. The compressive strength for clay-iron slag bricks shows an increase in compressive strength with an increase in percentage addition of iron slag. Compressive strength varies linearly with the addition of iron slag. Adding both fly ash and wood ash reduced the compressive strength of bricks compared to conventional brick. Compressive strength decreases linearly with an increase in the percentage of fly ash and wood ash. The density results are presented in Figure 6b. The addition of iron slag makes brick denser compared to conventional bricks, while the addition of fly ash and wood ash makes brick lighter. Radiation shielding of bricks is measured in term of ray absorption and linear attenuation coefficients, as presented in Figure 6c–f. It is evident from the results that iron slag is more effective in enhancing the ability of bricks to provide a shield against gamma radiation owing to its high density. On the other hand, addition of fly ash or wood ash has an adverse effect on the radiation-shielding ability of bricks. The linear attenuation coefficient varies linearly with the percentage addition of iron slag, wood ash, and fly ash. For all brick samples, higher shielding was observed for iron slug bricks at 25% replacement and lowest shielding was obtained for wood ash bricks at 20% replacement (Figure 6g). Figure 6h presents the relationship between density and linear attenuation coefficient. As evident from the available literature, a linear relationship is observed for linear attenuation and density in all specimens [15,24]. Because iron slag makes the brick denser, it enhances the shielding ability compared to conventional bricks. In contrast, fly ash and wood ash bricks have reduced shielding ability due to their lighter nature than normal bricks. It is evident that density is an essential factor of a material’s shielding ability against nuclear radiation. Shielding ability was also measured at different thicknesses, and a linear relation was observed. Shielding ability increases linearly with an increase in thickness. Gamma ray absorption followed an exponential trend, showing that a directly exposed surface contributes more by reducing the intensity of gamma rays.

### 3.2. Performance Evaluation of AI Models

**Figure 7:**
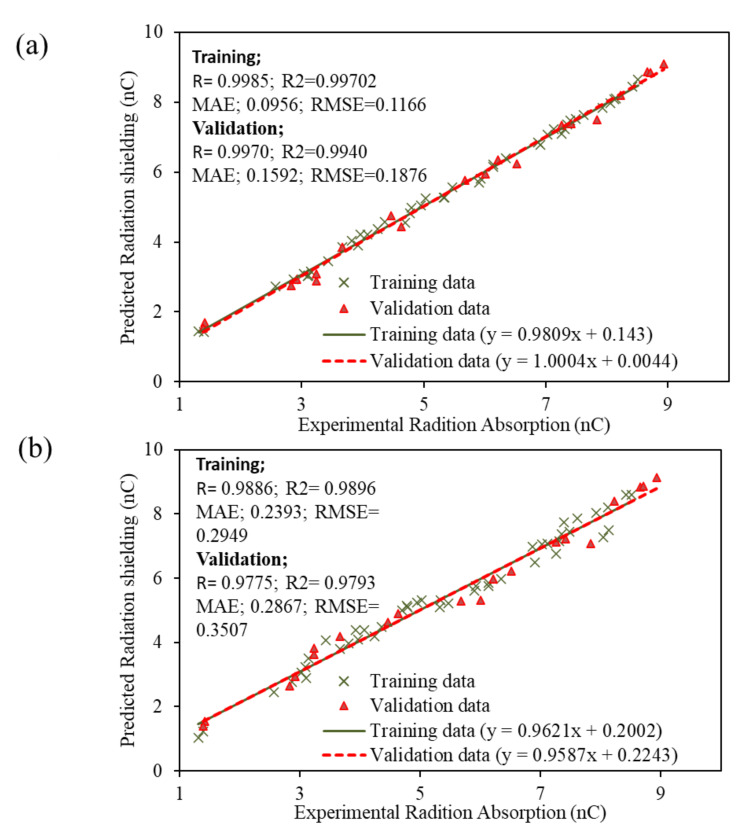
Comparison of experimental versus predicted results in the form of regression slopes and statistical indices: (**a**) ANN model, (**b**) GEP model.

The developed AI models were evaluated using statistical indices such as correlation coefficient (R), coefficient of determination (R^2^), mean absolute error (MAE), and root mean square error (RMSE) in accordance with the available literature [64,65,66,67,68,69,70,71,72]. R values obtained for the ANN model were 0.9985 and 0.9970 for the training and validation data, respectively. The values of R^2^ were observed as 0.99702 for the training set and 0.994005 for the validation sets of the ANN model (Figure 7a). The R values for the GEP model were observed as 0.9886 and 0.9775, with R^2^ values of 0.9896 and 0.9793, for the training and validation data, respectively (Figure 7b). MAE values of 0.09555 and 0.1592 nC for the ANN model and 0.2393 and 0.2867 nC for the GEP model were observed for training and validation data, respectively. Similarly, the observed values of RMSE were 0.116539 and 0.187575 for the ANN model and 0.2949 and 0.3507 for the GEP model for training and validation, respectively (Figure 7a,b). It is evident from the results that the R and R^2^ values are very close for the ANN and GEP models, which shows close agreement of experimental and predicted values. However, the error observed in the GEP model is greater compared to the error observed in the ANN model, which shows that ANN models show more robust performance than GEP models.

The performance of models was also measured in terms of regression slope because many researchers in the past have used the slope of the regression line for statistical evaluation [23,66]. To perform the regression analysis, the experimental results were plotted on the *x*-axis while the predicted results were plotted on the *y*-axis. Previous studies suggested that a regression slope of more than 0.80 indicates close agreement between experimental and predicted results. For the current study, regression analysis of the ANN model resulted in a slope of 0.9809 and 1.004 for training and validation data, respectively (Figure 7a). Similarly, the slope for the GEP model is 0.9621 and 0.9581 for training and validation data, respectively (Figure 7b). The regression analysis proves that the slope of the regression line for both models is close to an ideal slope, for which the slope is equal to 1. Regression models also show that the ANN model is relatively more accurate compared to the GEP model.

**Figure 8:**
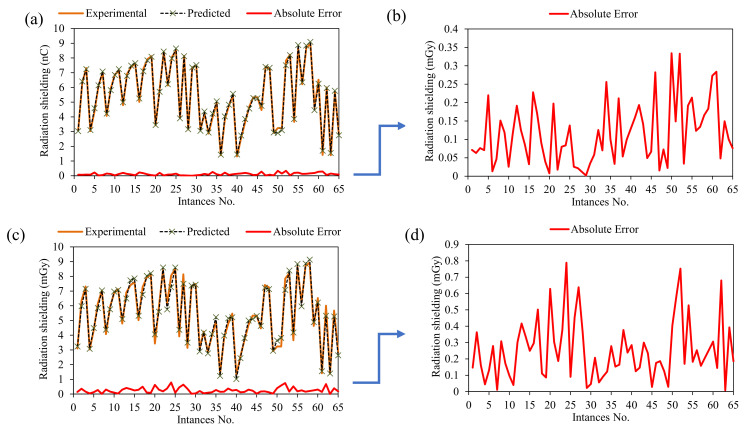
Error analysis of the developed models: (**a**) tracing of experimental by predictions for ANN model, (**b**) absolute error from ANN model, (**c**) tracing of experimental by predictions for GEP model, and (**d**) absolute error from GEP model.

The statistical assessment of ANN and GEP models was further enhanced by performing error analysis and tracing of experimental results with predictions made by the models, as presented in Figure 8a. The experimental results are traced very closely for both the models; however, the predictions made by the ANN more closely trace the experimental results compared to GEP. The error presented in Figure 8a,b ranges from 0 to 0.34 nC for the ANN model, while it ranges from 0 to 0.788 nC in the case of the GEP model. It is evident from the error analysis that the data points mostly converge about the zero line, with a maximum deviation of 0.34 and 0.788 nC in the ANN and GEP models, respectively.

It is evident from the above discussion that the ANN model has better performance compared to GEP; however, the GEP model has given acceptable results. GEP’s advantage over ANN lies in the fact that it generates a simple mathematical result that can be used for predicting a new dataset, unlike ANN, which has a black box nature. In the case of the ANN model, given data must be retained to predict a new dataset. Equations obtained from GEP modelling are given below as supplementary data in the form of a MATLAB model, which can be used for the prediction of radiation absorption by the bricks for the mentioned input variables.

### 3.3. Parametric Analysis

When it comes to AI-based modelling, it is crucial to carry out multiple assessments to make sure that the models are reliable and effective with various data combinations. Better training, validation, and testing results on current datasets are not a guarantee of the models’ overall superiority. The previous literature has suggested using parametric analysis, which is also used in the current study, to determine whether models are well trained and not just a correlation of inputs and output attributes. The variation in the output is plotted with the variation in one input variable over its entire range, while all the input parameters are fixed at their average value. All the input variables are separately repeated through the process.

**Figure 9:**
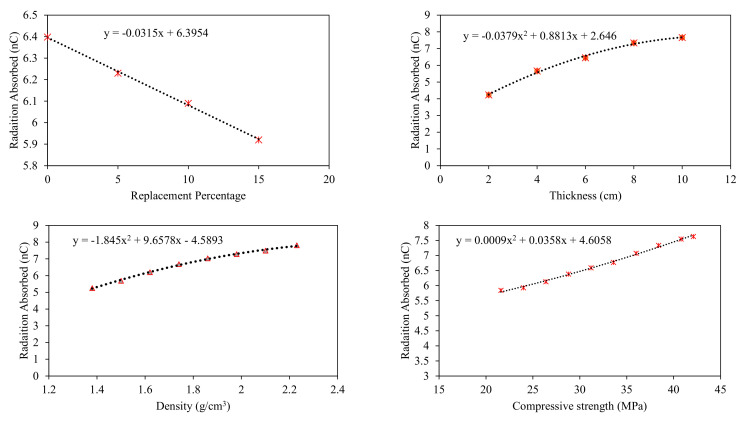
Parametric analysis of ANN model for Type 2 bricks.

**Figure 10:**
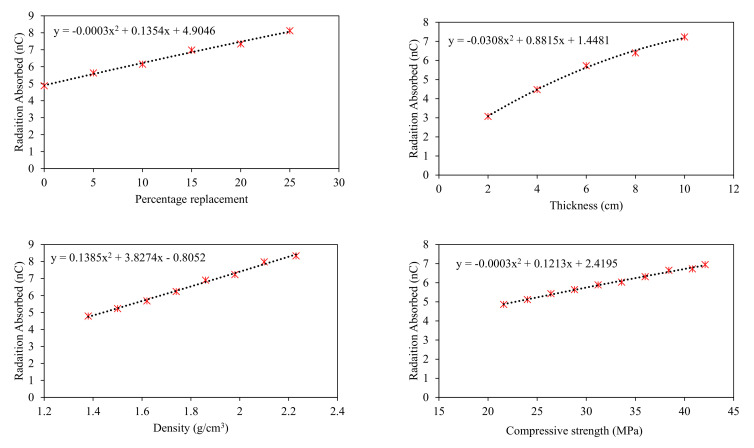
Parametric analysis of ANN model for Type 3 bricks.

**Figure 11:**
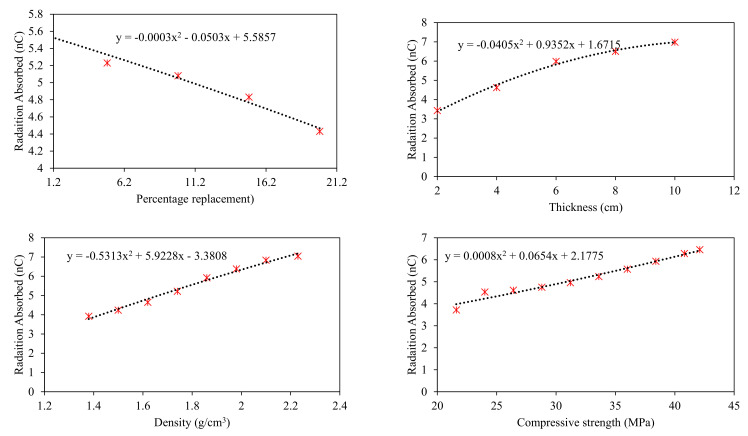
Parametric analysis of ANN model for Type 4 bricks.

Parametric analysis was performed to identify important variables affecting the target variable the most and depict the trend of input variables in contributing toward the target. A simulated dataset was developed by varying one input variable between its extreme values and keeping the other input variables constant at their average values. The simulated dataset was developed for clay-fly ash bricks (Type 2), clay-iron slag bricks (Type 3), and clay-wood ash bricks (Type 4). The simulated dataset was tested using the ANN model due to its superior performance. The analysis results are shown in Figure 9, Figure 10 and Figure 11 for the fly ash, iron slag, and wood ash bricks, respectively.

The results also show that thickness and density are the most influential parameters in enhancing the radiation-shielding ability in all types of bricks due to relatively greater change in compressive strength by changing these parameters. Among thickness and density, the thickness contributed more to enhancing the radiation shielding. Compressive strength also enhances radiation shielding, but the contribution is lower compared to thickness and density, as evident from Figure 9. The replacement percentage has no such direct relation with radiation shielding. Still, as it is affecting the density of bricks, in the case of iron slag, the percentage increase of iron slag enhances radiation shielding while the percentage replacement of fly ash and wood ash adversely affects the radiation shielding of bricks.

The parametric study further concluded that the maximum shielding of radiation occurred for the clay–iron slag brick at 25% replacement. In comparison, both fly ash and wood ash had reduced radiation shielding, and the minimum shielding was observed for wood ash bricks at 20% replacement. Gamma ray absorption revealed polynomial and linear variation with thickness and density, respectively. Compressive strength was also found to affect radiation shielding. Maximum radiation shielding was observed for the maximum compressive strength. From this research, we highly recommend considering these input parameters in the design of radiation shielding, especially in the design of radiation therapy chambers and nuclear power plants.

## 4. Conclusions

This study investigated the ability of modified clay bricks against the shielding capability against gamma rays. Conventional burnt clay bricks were modified using three different additives, namely fly ash, iron slag, and wood ash, as partial replacement for clay. The impact on radiation characteristics was studied at various thicknesses, densities, and compressive strengths. Additionally, ANN and GEP models were created to forecast the radiation resistance of bricks. The findings of this investigation were as follows:

- The two key elements that reduce the amount of gamma radiation are material density and brick thickness. The brick’s capacity to block radiation is enhanced by increasing its thickness and density. Among the investigated additives, it was observed that the addition of iron slag significantly increased the density, thus leading to improved resistance against radiation. The maximum radiation shielding was observed at 25% replacement of clay by iron slag. It was discovered that a rise in the compressive strength also improved the radiation capability of the bricks.
- The addition of fly ash and wood ash considerably decreased the density and compressive strength of modified clay bricks compared to the conventional bricks. The worst radiation capability of clay bricks was obtained at the maximum replacement of fly ash and wood ash investigated in the study.
- The AI models created for this study closely matched the outcomes of the experiments and the predictions. The ANN model that was created to forecast radiation shielding surpassed the GEP model. However, the GEP model’s importance may be seen in the straightforward mathematical relationship it generated since it can be used in the future to forecast how radiation shielding will affect fresh data without the use of a computer program.
- The results of the parametric analysis agreed with those of the experiment. The most important factors affecting the shielding capacity of concrete were discovered to be the thickness and density of clay bricks.

## Figures and Tables

**Table 1:** Summary of the previous literature concerning the use of bricks against radiation shielding.

Reference	Brick Type	Composition	Property Investigated
Mann et al. 2016 [37]	Clay–fly ash brick	Clay partially replaced with fly ash	Radiation shielding of brick
Mann, K.S. et al. 2016 [42]	Clay brick	Burnt clay brick collected from local brick factories in Punjab, India	Burnt clay bricks were investigated for surface storage facilities subjected to 0.001–15 MeV gamma ray photon energies
Escalera-Velasco, L.A. et al. 2020 [40]	Mexican artisanal bricks	Red clay bricks, yellow bricks, and bricks without cooking	Shielding behavior of artisanal bricks against ionizing photons
Kiatwattanacharoen et al. 2020 [43]	Barium sulphate bricks	Clay brick consists of barium sulphate	Clay bricks containing barium sulphate were investigated against X-ray radiation
Durak et al. 2022 [36]	Red and yellow clay-based bricks	Red and yellow clay-based bricks containing different amounts of Cobalt metal	Gamma and neutron shielding capacity of the brick
Velasco et al. 2022 [44]	Mexican artisanal bricks	Red clay bricks, yellow bricks and bricks without cooking	Radiation shielding parameters of bricks were investigated and compared with NBS concrete
Sidhu et al. 2022 [45]	Fly ash–lime–Gypsum (FaLG)	FaLG bricks are unfired compressed bricks consisting of flay ash, lime, and gypum	Shielding behavior of FaLG bricks was investigated

**Table 2:** Brick types and percentage addition of fly ash, wood ash, and iron slag.

S. No.	Brick Type	Material Added	Percentage Addition as a Replacement of Clay
1	Conventional Bricks (1)	No additional material	-
2	Clay–Fly Ash Bricks (2)	Fly ash	5%, 10%, 15%,
3	Clay–Wood Ash Bricks (4)	Wood ash	5%, 10%,15%, 20%
4	Clay–Iron Slag Brick (3)	Iron Slag	5%, 10%, 15%, 20%, 25%

**Table 3:** Physical properties of the constituent materials.

Material Property	Bulk Density (kg/m^3^)	Particle Specific Gravity	Color	Water Absorption
Clay	1680	2.35	Dark brown	-
Fly ash	1348	1.9	Black	-
Wood ash	1100	1.51	Light grey	-
Iron slag	2500	3.2	Dark grey	1.3

**Table 4:** Chemical composition of the constituent materials using XRF Analysis.

Chemical Composition	Clay	Fly Ash	Wood Ash	Iron Slag
SiO_2_	57%	41%	30.8%	27.56%
Al_2_O_3_	31%	22%	29%	4.24%
Fe_2_O_3_	7%	29%	2.34%	59.7%
MgO	3.5%	1%	8.98%	1.87%
CaO	1.5%	2%	11.23%	-
K_2_O	-	1.5%	12.13%	-
Na_2_O	-	1.81%	5.50%	-
MnO	-	-	-	2.23%
P_2_O_5_	-	-	-	2.45%
SO_3_	-	1.61%	-	1.90%
TiO_2_	-	-	-	-

**Table 5:** Grain size distribution of constituent materials.

Particle Type	Percent Finer				
	<20 μm	<50 μm	<75 μm	<100 μm	<150 μm
Clay	30	40	45	90	100
Fly ash	12	56	-	86	100
Wood ash	9	43	66	89	100
Iron slag	2	15	40	63	90

**Table 6:** Training dataset for model development.

Input Variables					Output Variable
Brick Type	Percentage Replacement	Thickness	Density	Compressive Strength	Gamma Ray Absorption
		(cm)	(g/cm^3^)	(MPa)	(nC)
2	5	2	1.68	34.42	3.0925
2	5	6	1.68	34.42	6.3425
2	5	8	1.68	34.42	7.3125
2	10	2	1.62	33.12	3.03
2	10	4	1.62	33.12	4.36
2	10	6	1.62	33.12	6.13
2	10	8	1.62	33.12	7.03
2	15	4	1.57	30.79	4.0725
2	15	6	1.57	30.79	5.9325
2	15	8	1.57	30.79	6.8725
2	15	10	1.57	30.79	7.1225
3	5	4	1.91	39.03	4.7925
3	5	6	1.91	39.03	6.9125
3	5	8	1.91	39.03	7.3925
3	5	10	1.91	39.03	7.6125
3	10	4	1.98	40.27	5.0225
3	10	6	1.98	40.27	7.2625
3	10	8	1.98	40.27	7.9225
3	10	10	1.98	40.27	8.1225
3	15	2	2.08	40.94	3.4325
3	15	4	2.08	40.94	5.9025
3	15	10	2.08	40.94	8.4195
3	20	4	2.14	41.2	6.13
3	20	6	2.14	41.2	8.05
3	20	8	2.14	41.2	8.51
3	25	2	2.23	42.1	3.92
3	25	6	2.23	42.1	8.14
1	0	2	1.76	36.8	3.14
1	0	8	1.76	36.8	7.35
1	0	10	1.76	36.8	7.48
4	5	4	1.51	29.32	3.1072
4	5	6	1.51	29.32	4.2427
4	10	4	1.47	26.4	2.8625
4	10	6	1.47	26.4	3.9625
4	10	8	1.47	26.4	4.9525
4	15	2	1.43	24.75	1.391
4	15	6	1.43	24.75	3.8125
4	15	8	1.43	24.75	4.7725
4	15	10	1.43	24.75	5.4625
4	20	2	1.38	21.6	1.31
4	20	4	1.38	21.6	2.57
4	20	6	1.38	21.6	3.66
4	20	8	1.38	21.6	4.7
4	20	10	1.38	21.6	5.33
4	5	8	1.51	29.32	5.3325

**Table 7:** Validation dataset for model development.

Brick Type	Percentage Replacement	Thickness	Density	Compressive Strength	Gamma Ray Absorption
		(cm)	(g/cm^3^)	(MPa)	(nC)
2	5	4	1.68	34.42	4.4625
2	5	10	1.68	34.42	7.4125
2	10	10	1.62	33.12	7.26
2	15	2	1.57	30.79	2.9146
3	5	2	1.91	39.03	3.2325
3	10	2	1.98	40.27	3.2325
3	15	6	2.08	40.94	7.8325
3	15	8	2.08	40.94	8.2225
3	20	2	2.14	41.2	3.66
3	20	10	2.14	41.2	8.66
3	25	4	2.23	42.1	6.22
3	25	8	2.23	42.1	8.71
3	25	10	2.23	42.1	8.93
1	0	4	1.76	36.8	4.63
1	0	6	1.76	36.8	6.52
4	5	2	1.51	29.32	1.4111
4	5	10	1.51	29.32	6.0025
4	10	2	1.47	26.4	1.3895
4	10	10	1.47	26.4	5.6725
4	15	4	1.43	24.75	2.8225

## Data Availability

The data used in this research have been properly cited and reported in the main text.

## Appendix A. The Code Developed for the ANN Model

x = ‘inputs’;
t = ‘RS’;
% Choose a training function
% For a list of all training functions type: help nntrain
% ‘trainlm’ is usually fastest.
% ‘trainbr’ takes longer but may be better for challenging problems.
% ‘trainscg’ uses less memory. Suitable in low memory situations.
trainFcn = ‘trainlm’; % Levenberg–Marquardt backpropagation.
% Create a fitting network
hiddenLayerSize = 10;
net = fitnet(hiddenLayerSize,trainFcn);
% Setup division of data for training, validation, testing
net.divideParam.trainRatio = 70/100;
net.divideParam.valRatio = 30/100;
% Train the network
[net,tr] = train(net,x,t);
% Test the network
y = net(x);
e = gsubtract(t,y);
performance = perform(net,t,y)
% View the network
view(net)
% Plots
% Uncomment these lines to enable various plots.
%figure, plotperform(tr)
%figure, plottrainstate(tr)
%figure, ploterrhist(e)
%figure, plotregression(t,y)
%figure, plotfit(net,x,t)
